# Embolic Stroke of Unknown Source in a Young Patient due to Pulmonary Arteriovenous Malformation Masquerading as a Patent Foramen Ovale

**DOI:** 10.1016/j.case.2026.03.006

**Published:** 2026-05-13

**Authors:** Micah J. Eimer, Seth Klein

**Affiliations:** a Division of Cardiology, Bluhm Cardiovascular Institute, Feinberg School of Medicine, Northwestern University, Chicago, Illinois; b Division of Interventional Radiology, Bluhm Cardiovascular Institute, Feinberg School of Medicine, Northwestern University, Chicago, Illinois

## Abstract

• PAVMs and HHT should be considered in young patients with ESUS.
• PAVMs can be difficult to differentiate from PFOs by echocardiography.
• CT can noninvasively diagnose PAVMs.
• ASC during TTE can diagnose and help quantify shunt size in HHT.

## Graphical abstract

**Figure:**
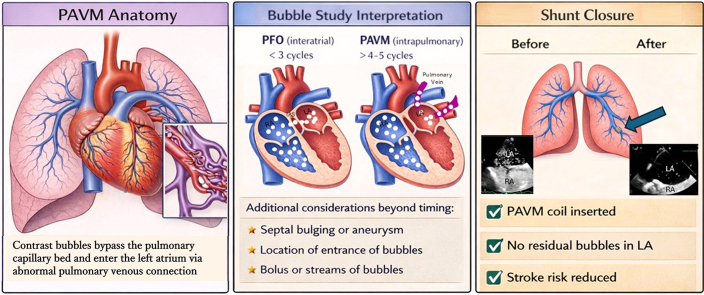

## Introduction

Pulmonary arteriovenous malformations (PAVMs) are an uncommon cause of embolic stroke of unknown source (ESUS).^1^ Pulmonary arteriovenous malformations are usually diagnosed during a transthoracic echocardiogram with agitated saline contrast (ASC) study that shows bubbles appearing after greater than 3 cardiac cycles in the left heart. Discrimination of interatrial shunts (patent foramen ovale [PFO]) from transpulmonary (PAVM) right-to-left shunts can be difficult at times as the bubble study and maneuver quality are variable and large PAVMs can rapidly fill the left atrium (LA) with bubbles. However, the differentiation is critical as the approach to recurrent stroke prevention is completely different among the 2 clinical scenarios.

## Case Presentation

A 52-year-old female patient with a remote history of deep venous thrombosis and pulmonary embolism underwent a craniotomy for resection of a meningioma. Forty-eight hours prior to the craniotomy the patient underwent prophylactic placement of an infrarenal inferior vena cava (IVC) filter. On postoperative day 2, the patient reported acute left-sided weakness, and neurologic imaging revealed multiple acute right-sided cerebral infarctions in multiple territories consistent with embolic source.

**Figure 1:**
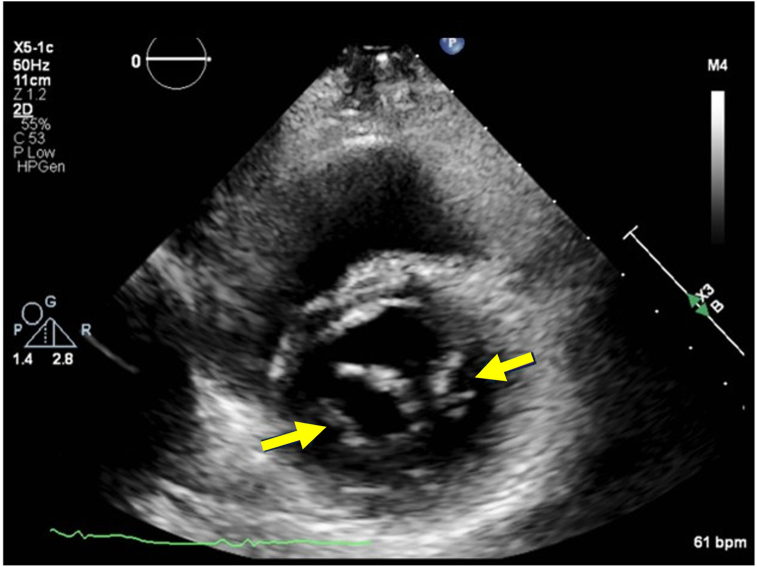
Two-dimensional transthoracic echocardiogram, basal parasternal short-axis diastolic view, demonstrates 2 separate mitral orifices *(arrows)* consistent with a nonstenotic, congenital double orifice MV.

Workup at that time showed no evidence of a hypercoagulable state, no atrial fibrillation on inpatient or extended ambulatory event monitor, and no thrombus in the deep veins of the leg or at the site of the IVC filter. There was no significant atherosclerosis of the aortic arch or carotid arteries. A transthoracic echocardiogram done preoperatively showed a normal ejection fraction and a double-orifice mitral valve (MV; Figure 1) without evidence of left atrial enlargement or significant MV dysfunction. An ASC study with a Valsalva maneuver was abnormal and thought to represent an interatrial shunt (Video 1) based on the appearance of bubbles in the LA within 3 cardiac cycles.

A transesophageal echocardiogram (TEE) done on postoperative day 3 (Video 2) showed no intracardiac thrombus and confirmed the strongly positive ASC study with bubbles appearing on the left side within 3 cardiac cycles. On TEE there was no evidence of interatrial communication by color-flow Doppler and bubbles were not visualized traversing a PFO.

**Figure 2:**
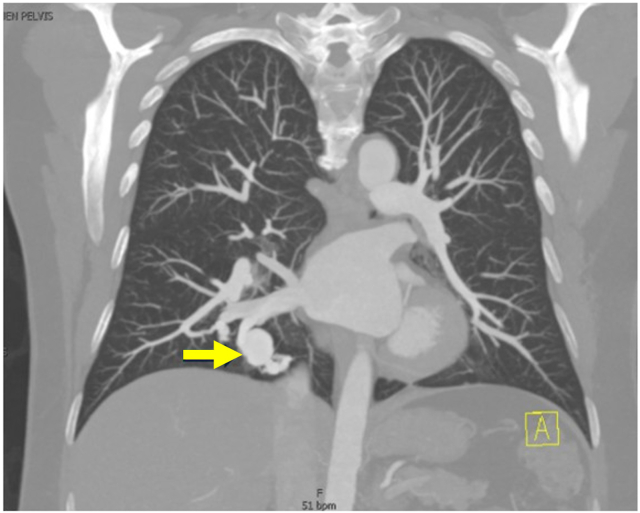
Chest CT angiography, maximal intensity projection, coronal display, demonstrates dilated peripheral pulmonary blood vessels in the right lower lobe *(arrow)* suggestive of a PAVM.

**Figure 3:**
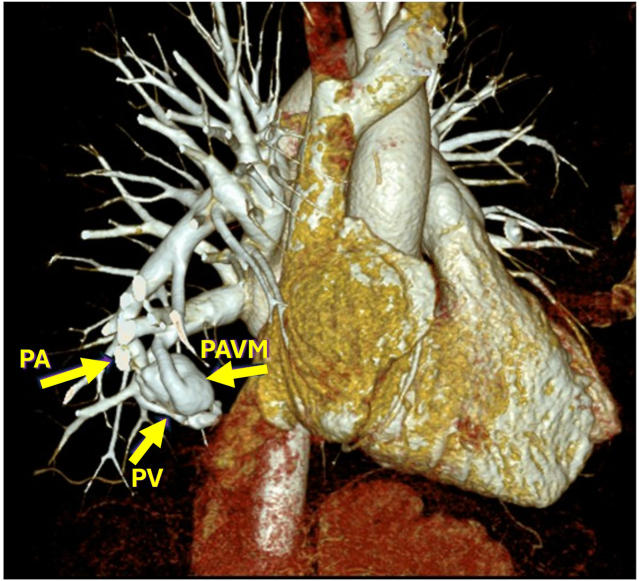
Chest CT angiography, three-dimensional volume-rendered reconstruction, anterior projection, demonstrates a complex PAVM with multiple hypertrophied feeding pulmonary artery branches (PA) and several draining pulmonary veins (PV).

Review of the patient's chart revealed a computed tomography (CT) of the chest done preoperatively in concert with evaluation of the brain mass. The CT demonstrated a lobulated lesion of the medial right lower lobe measuring 2.3 × 2.1 cm, which was suggestive of a vascular malformation (Figure 2, Figure 3).

**Figure 4:**
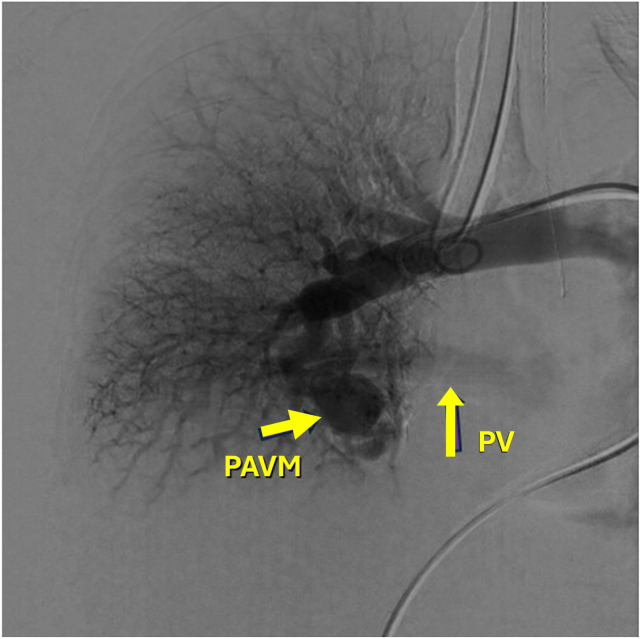
Invasive, selective pulmonary angiogram performed prior to placement of a percutaneous closure device, zoomed arterial phase display, demonstrates the right pulmonary artery after contrast injection with abnormal early filling of the right lower lobe pulmonary vein through the PAVM.

**Figure 5:**
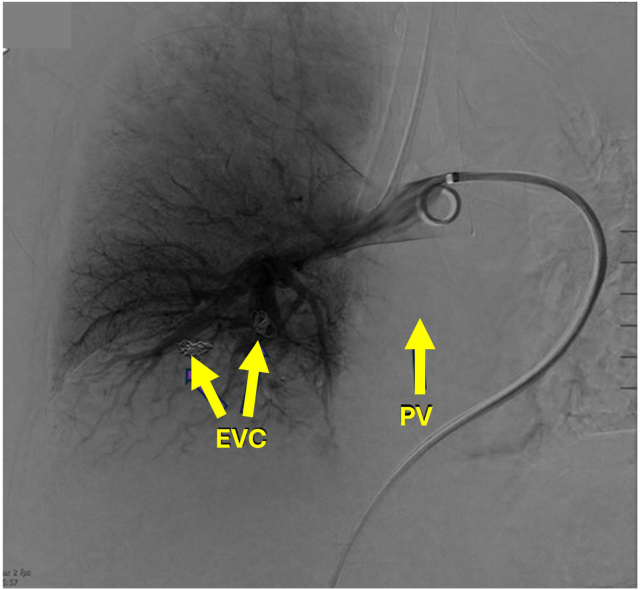
Invasive, selective pulmonary angiogram performed after placement of a percutaneous closure device, zoomed arterial phase display, demonstrates the right pulmonary artery after contrast injection and resolution of the early filling of the pulmonary vein, consistent with successful PAVM occlusion. *EVC,* Endovascular coil; *PV,* pulmonary vein.

The patient underwent selective pulmonary angiography, which revealed a significant right lower lobe PAVM (Figure 4), which was coiled using percutaneous closure devices (Figure 5).

A follow-up TEE was performed that showed a small right-to-left shunt in a pattern more consistent with a residual (or additional) typical PAVM (Video 3). The patient continues to undergo annual surveillance echocardiography with ASC studies, and the IVC filter was removed.

## Discussion

The workup of ESUS includes an ASC study to screen for right-to-left shunting, either intracardiac (PFO) or intrapulmonary (PAVMs). Differentiation between the two can be difficult as in this case large PAVMs with high flow can produce bubbles in less than 3 to 4 cardiac cycles on the left side particularly during periods of increased cardiac output or positioning that increases PAVM flow.^2^ While PAVMs are less prevalent in the population compared to PFOs, the ability of a PAVM to cause a stroke may be considerably higher based on the continuous right-to-left flow and the frequent occurrence of multiple PAVMs in the same patient.^3^

The data on the ability of PAVMs to cause stroke are best demonstrated in the hereditary hemorrhagic telangiectasia (HHT) literature.^4^ In a study of patients with HHT, the prevalence of clinical stroke is as high as 38%, while the prevalence of silent strokes is 55%. The risk of stroke in patients with PAVM is predicted by the shunt size as assessed by ASC study.^5^ The strokes in patients with PAVMs are also more impactful as the patients tend to be younger and healthier. The prognosis from stroke is worse in patients with PAVMs, who are more likely to have multifocal infarcts, suffer from more cerebral edema, aphasia, and hemiplegia, and have more bleeding and clotting complications.^6^

The role of PAVMs in ESUS outside of HHT is not well-known. Agitated saline contrast studies that are positive for transpulmonary shunting are rarely followed up with a chest CT, which is the screening test of choice for PAVMs. However, it seems reasonable that in young patients with ESUS and a positive bubble study not convincingly related to a PFO on TEE a search for a PAVM be undertaken in an attempt to prevent recurrent stroke and avoid inappropriate PFO closure device placement. Innovation is needed in this area to better differentiate PFO versus PAVM.

The double-outlet MV is thought to be an incidental finding in this case as there was no hemodynamic significance and the LA was not enlarged. The IVC filter may have been contributory as increased risk of deep vein thrombosis and thrombus formation on the device itself are known complications of IVC filter placement.^7^

## Conclusion

When evaluating patients with ESUS, differentiation of PAVM from PFO is critically important and guides the future intervention to prevent recurrent stroke. The standard dogma of timing bubble appearance in the left heart to cardiac cycles is flawed. Clinicians should appreciate that large PAVMs can mimic PFOs on echocardiography due to rapid pulmonary transit. Consideration of other factors such as the relationship to maneuvers, bolus versus continuous appearance of bubbles, and visualization of bubbles in pulmonary veins can be helpful.

## Consent Statement

The authors declare that since this was a non-interventional, retrospective, observational study utilizing de-identified data, informed consent was not required from the patient under an IRB exemption status.

## Ethics Statement

The authors declare that the work described has been carried out in accordance with The Code of Ethics of the World Medical Association (Declaration of Helsinki) for experiments involving humans.

## Declaration of Generative AI and AI-assisted Technologies in the Manuscript Preparation Process

During the preparation of this work the author(s) used Chat GPT in order to create graphics for the graphical abstract. After using this tool/service, the author(s) reviewed and edited the content as needed and take(s) full responsibility for the content of the published article.

## Disclosure Statement

The authors reported no actual or potential conflicts of interest relative to this document.

## Supplementary Data

Video 1 Two-dimensional transthoracic echocardiogram, apical 4-chamber view after injection of ASC and Valsalva maneuver, demonstrates normal left ventricular size and function, normal left atrial size, and bubbles within the left heart, consistent with a right-to-left shunt of unknown etiology.

Video 2 Two-dimensional TEE, midesophageal, rightward rotated long-axis (0°) view of the atrial septum during ASC study, performed prior to placement of a percutaneous closure device in the PAVM, demonstrates multiple bubbles continuously entering the LA prior to the third cardiac cycle, raising the suspicion for a PFO.

Video 3 Two-dimensional TEE, midesophageal, long-axis (55°) view of the atrial septum during ASC study and then a rotational cine display (162° to 0°) of the complete atrial septal anatomy, performed after placement of a percutaneous closure device in the PAVM, demonstrates a few bubbles in the LA that appear late, consistent with a small residual PAVM shunt.
